# 

**Published:** 1902-03

**Authors:** 


*THE BRISTOL MEDICO-CHIRURGICAL SOCIETY.*

### CONTENTS.

| | PAGE |
|---|---|
| **The Reputation of the Hotwells (Bristol) as a Health-Resort.** By L. M. GRIFFITHS, M.R.C.S. | 1 |
| **On Some Urinary Infections, with especial reference to their Treatment by Urotropin.** By J. ODERY SYMES, M.D. Lond., D.P.H. | 27 |
| **A Retrospect of a Further Series of Intra-Abdominal Operations.** By JAMES SWAIN, M.S., M.D. Lond., F.R.C.S. Eng. | 33 |
| **The Use of X-Rays in the Diagnosis of Renal Calculi.** By JAMES TAYLOR, M.R.C.S. | 44 |
| **Notes on a Case of Variola Nigra. (Malignant or Black Smallpox.)** By D. S. DAVIES, M.D. Lond. | 49 |
| **Notes on Ethyl Chloride as a General Anæsthetic.** By FRANK H. ROSE, M.R.C.S., L.R.C.P. (Lond.) | 53 |
| **A Case of Gastro-Jejunostomy for Cancer of the Pylorus.** By J. PAUL BUSH, C.M.G., M.R.C.S. | 57 |
| **Progress of the Medical Sciences:** | |
| **Medicine.** THEODORE FISHER, M.D. Lond. | 61 |
| **Surgery.** J. LACY FIRTH, M.D., B.S. Lond., F.R.C.S. Eng. | 65 |
| **Reviews of Books** | 70 |
| **Editorial Notes** | 76 |
| **Notes on New Drugs and Preparations for the Sick** | 82 |
| **The Library of the Bristol Medico-Chirurgical Society** | 83 |
| **Meetings of Societies** | 85 |
| **Local Medical Notes** | 94 |

